# Elevations of Cardiac Troponin in Patients Receiving Immune Checkpoint Inhibitors: Data From a Prospective Study

**DOI:** 10.1016/j.jacadv.2024.101375

**Published:** 2024-11-07

**Authors:** Pieter F. van den Berg, Valentina Bracun, Michel Noordman, Peter van der Meer, Canxia Shi, Sjoukje F. Oosting, Joseph Pierre Aboumsallem, Sanne de Wit, Wouter C. Meijers, Mathilde Jalving, Michel van Kruchten, Rudolf A. de Boer

**Affiliations:** a Department of Cardiology, University Medical Center Groningen, Groningen, the Netherlands; b Department of Medical Oncology, University Medical Center Groningen, Groningen, the Netherlands; c Erasmus Medical Center, Cardiovascular Institute, Thorax Center, Department of Cardiology, Rotterdam, the Netherlands

**Keywords:** cardiac biomarkers, hs-TnT, hypertroponinemia, ICI, immunotherapy, myocarditis

## Abstract

**Background:**

Immune checkpoint inhibitors (ICIs) are increasingly used in the treatment of cancer. However, immune-related adverse events are prevalent in patients receiving ICI therapy. A serious immune-related adverse event is ICI-myocarditis, which is complex to diagnose given that the significance of early symptoms and biomarker trajectories, such as high-sensitivity troponin T (hs-TnT) are unclear.

**Objectives:**

The purpose of the study was to evaluate kinetics of hs-TnT in cancer patients receiving ICI and to identify patients at risk of developing ICI-myocarditis.

**Methods:**

This prospective, observational, single-center study included 164 patients receiving ICI therapy. Patients’ history, demographics, and clinical characteristics, as well as survival statistics, were collected from electronic patient records and used to analyze associations between elevated hs-TnT (≥14 ng/L) and a significant rise in hs-TnT (100% rise from baseline, with an absolute value ≥2x upper limit of normal (ie, ≥28 ng/L) with ICI-myocarditis.

**Results:**

We included 164 patients with a mean follow-up time of 1.60 ± 0.91 years. Melanoma was the most common type of cancer in the patient population, and most patients received treatment with programmed cell death protein 1 (PD-1). Twenty-six patients (16%) exhibited significant hs-TnT elevations, while 8 patients (5%) developed ICI-myocarditis. In 18 of 26 (69%) patients, ICI-myocarditis could not be diagnosed with certainty, while 10 of 26 (38%) patients had no other signs of symptoms of cardiac damage. All 8 myocarditis cases were preceded by significantly higher hs-TnT elevations than asymptomatic patients. Despite a high ICI-myocarditis incidence in our study population, cardiac mortality remained low (4%).

**Conclusions:**

Significant hs-TnT elevations occur more often than previously reported, are often asymptomatic, and do not always lead to myocarditis diagnosis.

Immune checkpoint inhibitors (ICIs) have revolutionized cancer treatment, rapidly increasing their use in both metastatic disease and the (neo)adjuvant setting.^1^, ^2^, ^3^ However, the reactivation of the immune system with ICIs carries the risk of immune-related adverse events (irAEs), some of which can be life-threatening.^4^^,^^5^ Adequate recognition and management of irAEs are therefore crucial, especially since the use of ICIs continues to rise.^6^ Fulminant ICI-myocarditis poses a diagnostic challenge due to overlapping clinical presentations with various cardiovascular (CV) complications (such as heart failure, acute coronary syndrome, and arrhythmias) and cancer-related complications (including anemia and cachexia).^7^, ^8^, ^9^, ^10^, ^11^, ^12^ Currently, the available diagnostic modalities, including cardiac biomarkers, electrocardiogram (ECG), and cardiac imaging (transthoracic echocardiogram [TTE] and cardiovascular magnetic resonance imaging [cMRI]), have limited sensitivity and specificity.^10^^,^^11^ Biomarkers, specifically high-sensitivity troponin T (hs-TnT), play a pivotal role in diagnosing CV complications during ICI treatment, mainly ICI-myocarditis.^13^ For that reason, understanding the dynamics of hs-TnT levels during ICI treatment is of paramount importance.^14^ The issue of elevations in hs-TnT in ICI myocarditis has recently received attention.^15^ The frequency of hs-TnT changes during ICI treatment and the appropriate cut-off value to suggest cardiac adverse events remain unclear. Elucidating the temporal patterns and magnitude of hs-TnT changes during ICI treatment could aid in identifying patients at higher risk of developing ICI-related cardiac complications, facilitating early intervention, and preventing life-threatening outcomes. At the same time, establishing optimal cut-off values for hs-TnT as a screening tool could enhance its specificity and clinical utility in distinguishing true cardiac events from false-positive results, thereby optimizing patient management and reducing unnecessary interventions.

In order to address these knowledge gaps and to assess the value of hs-TnT as a screening tool during ICI treatment for patients at risk of developing ICI-myocarditis, we conducted a prospective analysis of serial hs-TnT levels in patients receiving ICI therapy.

## Methods

### Study design and population

We conducted a prospective, observational, single-center study with patients older than 18 years, diagnosed with cancer and treated with ICI (at least 1 cycle) between October 2019 and February 2022. Patients were eligible for enrollment if they had baseline hs-TnT measured within 10 days before the first ICI treatment and at least 1 follow-up hs-TnT measurement during treatment (Supplementary Figure 1). Due to a different screening protocol and therefore lack of baseline hs-TnT, only 2 patients with lung cancer were included in the study. The study complied with the Declaration of Helsinki and was approved by the central ethics committee of our center (Research Registration Number 202100884), who waived the necessity to obtain informed consent.

### Assessment of covariates and definitions

Patient characteristics, physical examination, CV and oncological history, tumor and treatment characteristics, treatment response, plasma biomarker values, TTE, ECG, the occurrence of irAE, survival statistics, and the moment of data retrieval (ie, at the latest May 2023) were collected as reported in the electronic patient chart. Cause of death (defined as either due to progression of cancer, due to CV disease, or as all-cause mortality in absence of proof for the former two), which was discussed by a panel of 1 clinical oncologist and 1 cardiologist in case of unclarity; as survival endpoint, all-cause mortality was considered as endpoint. An irAE was defined as any described immune-related side-effect of immune checkpoint therapy that mimics an autoimmune response and is able to manifest in almost the entire body. The occurrence of an irAE was determined by the treating oncologist and obtained from the electronic patient chart. If patients received multiple, consecutive ICI treatments, or when ICI monotherapy was preceded by combination therapy, the first treatment regimen was used for the analysis. Body mass index (BMI) was calculated as weight divided by height squared (kg/m^2^). Stable coronary artery disease was defined as coronary artery stenosis ≥50% that was diagnosed by either coronary computed tomography scan or cardiac catheterization. Significant valve dysfunction included moderate and severe valve abnormalities. Active smoking was defined as current smoking or smoking within the previous year. *Elevated hs-TnT* was defined as the 99th percentile of the distribution of troponin levels in the healthy reference population (a value above 14 ng/L).^16^^,^^17^ We defined a *significant rise* in hs-TnT as a 100% rise from baseline, with an absolute value ≥2x upper limit of normal (ULN) (ie, ≥28 ng/L). If patients with a *significant rise* had multiple measurements that fit the definition of a *significant rise*, the highest hs-TnT value was recorded and considered in our analysis. We chose a doubling of the hs-TnT as a threshold for an abnormal finding, as it is a common threshold in the biomarker literature and already minor changes in hs-TnT have important diagnostic and prognostic consequences.^18^^,^^19^ The first step in patients with a *significant* hs-TnT rise was to evaluate whether diagnoses other than ICI-myocarditis (eg, pulmonary embolism, renal insufficiency, and acute coronary syndrome) could explain the hs-TnT rise. If so, the possibility of ICI-myocarditis was discarded. If there was no satisfactory alternative explanation for the hs-TnT rise, clinical signs and symptoms were collected with ECG and TTE findings and cMRI to ascertain the definite likelihood of ICI-myocarditis. To this end, we used previously published hierarchical definition of ICI-myocarditis based on the major and minor criteria and assigned myocarditis probability as *low* probability, *high* probability, or *definite* myocarditis (Table 1).^20^ Therefore, a definitive diagnosis of ICI-myocarditis was established when a patient exhibited a *significant* elevation in hs-TnT, accompanied by 1 major and at least 1 minor criteria. The major criterion relied on cMRI in accordance with the Lake Louise criteria for myocarditis, or the presence of noteworthy T2, T1, myocardial extracellular volume, or late gadolinium enhancement changes indicative of suspected myocarditis. Minor criteria include: 1) symptoms of cardiac dysfunction (new increase in NYHA functional class); 2) ECG changes (new arrhythmia or conduction disturbances); 3) TTE changes (≥5% drop in left ventricular ejection fraction, with or without new wall motion abnormalities); 4) presence of other irAE; 5) suggestive but nondiagnostic cMRI findings. Asymptomatic hs-TnT elevations (*ie, hypertroponinemia*), in the absence of other criteria and in the absence of other explanations for hs-cTn rise, were defined as a *low ICI-myocarditis probability*. In contrast, hs-TnT elevations accompanied by minor criteria but insufficient to classify as definite ICI-myocarditis received a *high* myocarditis probability. If myocarditis diagnosis and/or cause of death were uncertain, a consultation between cardiologist and oncologist ensued to collaboratively determine the diagnosis and most appropriate course of action. The first date that mentioned ICI-myocarditis (either by cardiologists or treating physicians or cMRI findings), when the diagnosis fulfilled the criteria, was taken.

**Table 1:** Diagnosis of ICI-Myocarditis Based on the Presence of Clinical Signs and Symptoms of Cardiac Dysfunction

	Excluded (n = 138)	Low (n = 10)	High (n = 8)	Definitive (n = 8)
Hs-TnT, ng/L	13 (8-20)	40.5 (36-94)	104 (61.5-136)	242 (168-1,108.5)
Minor criteria				
cMRI				

Values are median (IQR) unless otherwise indicated. All patients had to have a significant Hs-TnT elevation. Patients in which a Hs-TnT rise could be attributed to another diagnosis than ICI-myocarditis were excluded. Higher Hs-TnT levels are associated with an increased likelihood of ICI-myocarditis.

Minor criteria include symptoms, ECG changes, TTE changes, other irAE, or nondiagnostic cMRI.

cMRI = cardiac magnetic resonance imaging; ECG = electrocardiogram; ICI = immune checkpoint inhibitor; hs-TnT = high-sensitive troponin T; TTE = transthoracic echocardiography.

### Data collection and biomarker measurements

Blood was drawn, and biomarkers, including hs-TnT, were measured at each treatment visit where new dose was administered (every 3-4 weeks), starting from treatment initiation. A total of 1,598 hs-TnT measurements were conducted. Of these, 164 were baseline measurements, while 1,434 measurements were conducted during the treatment. In case of a (suspected) ICI-myocarditis, additional values of hs-TnT were added on the date of hospitalization, date of visit at the cardio-oncologic outpatient clinic, or the date of cMRI. The frequency of hs-TnT measurements per individual patient spanned a range from 2 to 35, resulting in a mean value of 8.74 measurements per patient. The shortest interval between 2 ICI treatments was for anti-PDL1 treatment, which is dosed every 2 weeks. This comprised 15.8% of the patients. All other patients had an interval of 3 or 4 weeks: anti-PD1, combination anti-PD1/anti-CTLA4, and anti-PD1 + TKI were dosed every 3 weeks, and anti-PD1 treatment every 4 weeks. Cardiac biomarkers were measured using Roche Elecsys assay using standard methods (Roche Diagnostics GmbH) and extracted from the electronic patient record at each instance of ICI-treatment with a window of ±3 days. In the case of a (suspected) ICI-myocarditis, additional values of hs-TnT were added on the date of hospitalization, date of visit at the cardio-oncologic outpatient clinic, or the date of cMRI. Values of other plasma biomarkers were collected when they were not older than 30 days of the first ICI dose or 10 days of any following ICI treatment.

### Statistical analysis

Data are presented based on skewness of their distribution; symmetrically distributed variables are presented as mean ± SD, and skewed data are presented as median (IQR). Categorical data are presented as absolute numbers (N, %). For human data, percentages and years are rounded to a whole number. In case of skewed distribution, measurements were log-transformed for analyses. Differences between groups were estimated using 1-way analysis of variance, Kruskal-Wallis or chi-squared test where appropriate. Survival in subgroups was evaluated using the Simon-Makuch, where time variable of 4 weeks was chosen. The multivariable regression model for all-cause mortality was based on a directed acyclic graph (Supplementary Figure 2). A *P* value of ≤0.05 was considered statistically significant. We acknowledge that the choice of a 0.05 significance level as a cutoff for statistical significance was arbitrary. Therefore, it is essential to interpret the results presented in this study with caution. All statistical analyses were performed using STATA/SE16 (StataCorp) and R (R Foundation for Statistical Computing).

## Results

### Baseline patient characteristics

In the final analysis, we included 164 patients, of whom 63% were male, with a medium age of 65 ± 12 years (Table 2). Patients were slightly overweight with BMI of 25.9 ± 5.2 kg/m^2^, and they had a blood pressure of 138/80 mm Hg (systolic blood pressure ±22; diastolic blood pressure, IQR: 70-90 mm Hg). Median hs-TnT was 10.5 ng/L (IQR: 6.5-15 ng/L) and median N-terminal prohormone of B-type natriuretic peptide was 195 ng/L (IQR: 53-467 ng/L). Estimated glomerular filtration rate of 85 ml/min/1.73 m^2^ (IQR: 68-94 ml/min/1.73 m^2^), hemoglobin (Hb) of 8.1 mmol/L (IQR: 6.9-8.9 mmol/L), and leukocytes of 7.6 mmol/L (IQR: 6.0-10.0 mmol/L) were all found to be within the normal range. Melanoma was the most commonly diagnosed cancer (59%), followed by renal cell carcinoma (18%) and bladder cancer (6%). A majority of patients received PD-1 treatment (43%), followed by either ICI-combination (PD[L]-1 and CTLA-4) therapy or monotherapy with PD-L1 (17%). While most patients (71%) had no history of CV disease, most of them (73%) had at least 1 risk factor, of which smoking history (33%) and hypertension (17%) were most prevalent.

**Table 2:** Baseline Characteristics of Patient Population (N = 164)

Patient demographics	
Sex	
Male	104 (63)
Female	60 (37)
Age, y	63 ± 12
BMI, kg/m^2^	25.9 ± 5.2
SBP, mm Hg	138 ± 22
DBP, mm Hg	80 (70-90)
Heart rate, beats/min	84 (67-94)
Laboratory parameters	
Hs-TnT, ng/L	10.5 (6.5-15)
NT-proBNP, ng/L	195.5 (53-467)
eGFR, mL/min/1,73 m^2^	85 (68-94)
Hb, mmol/L	8.1 (6.9-8.9)
Leucocytes, 10^9^/L	7.6 (6-10)
Oncological demographics	
Type of cancer	
Renal	30 (18)
Melanoma	97 (59)
Lung	2 (1)
Head and neck	8 (5)
Bladder	10 (6)
Merkel cell	2 (1)
Squamous cell skin	7 (4)
GI	8 (5)
Cancer treatment	
PD-1	70 (43)
PDL-1	27 (17)
PD/PDL-1 + CTLA4	58 (35)
ICI + TKI	7 (4)
ICI + other	1 (1)
CV history	
No	116 (71)
ACS	4 (3)
Stable CAD	2 (1)
Significant valve dysfunction	4 (3)
Atrial fibrillation/atrial flutter	7 (4)
Other atrial arrhythmias	1 (1)
Ventricular arrhythmias	1 (1)
DVT/PE	12 (7)
TIA/CVA	8 (5)
Peripheral vascular disease	3 (2)
Conduction disturbance	5 (3)
Risk factors	
Active smoking	11 (7)
Hypercholesterolemia	2 (1)
Hypertension	28 (17)
Diabetes mellitus type 2	14 (9)
Family history for CVD	10 (6)
Smoking history	54 (33)
No	32 (20)
Unknown	12 (7)
CV medication	
Beta-blocker	3 (2)
ACEI/ARB	7 (4)
Calcium-blocker	10 (6)
Statin	10 (6)
VKA/NOAC/LMWH	14 (9)
Acetylsalicylic acid	15 (9)
Diuretic	8 (5)
Other	14 (9)
No	83 (51)

Values are n (%), mean ± SD, or median (IQR).

ACS = acute coronary syndrome; ACEI = angiotensin converting enzyme inhibitor; ARB = angiotensin receptor blocker; BMI = body mass index; CAD = coronary artery disease; CV = cardiovascular; CVA = cerebrovascular accident; DBP = diastolic blood pressure; DVT = deep venous thromboembolism; eGFR = estimated glomerular filtration rate; Hb = hemoglobin; hsTnT = high-sensitivity troponin T; LMWH = low molecular weight heparin; NOAC = novel oral anticoagulant; NT-proBNP = N-terminal prohormone of B-type natriuretic peptide; PE = pulmonary embolism; SBP = systolic blood pressure; TKI = tyrosine kinase inhibitor; TIA = transient ischemic attack; VKA = vitamin K antagonist.

### Hs-TnT trajectories and ICI-myocarditis incidence

Baseline hs-TnT values were elevated (ie, ≥14 ng/L) in 58 patients (35%), while 106 patients (65%) presented with normal baseline hs-TnT values. During study follow-up, 95 patients (58%) presented with elevated hs-TnT levels, of whom 39 patients (24%) had normal baseline hs-TnT levels, and 56 patients (34%) had already elevated hs-TnT levels at baseline (Table 3). When using a more stringent, arbitrary cut-off of a doubling in hs-TnT value and an absolute value of at least 2 × ULN, 26 patients (16%) developed a *significant* elevation in hs-TnT.

**Table 3:** hs-TnT Levels During Study Follow-Up

hs-TnT baseline <14, hs-TnT max <14	67 (41)
hs-TnT baseline <14, hs-TnT max ≥14	39 (24)
hs-TnT baseline <14, hs-TnT max ≥28	14 (8)
hs-TnT baseline >14, hs-TnT max <14	2 (1)
hs-TnT baseline >14, hs-TnT max ≥14	56 (34)
hs-TnT baseline >14, hs-TnT max ≥28	26 (16)
hs-TnT max ≥2x hs-TnT base	26 (16)

Values are n (%). Number of patients described by their hs-TnT status at baseline and during the ICI treatment.

hs-TnT = high-sensitive troponin T; ICI = immune checkpoint inhibitor; max = maximum.

Despite the large number of *significantly* elevated hs-TnT, only 8 patients (5%) could be diagnosed with definite ICI-myocarditis diagnosis, according to the diagnostic criteria. Meanwhile, 8 patients (5%) presented with a high ICI-myocarditis suspicion defined by presence of both significant hs-TnT change and at least 1 minor criterion that were insufficient to definitely diagnose ICI-myocarditis. Finally, 10 patients (6%) had significantly elevated hs-TnT without any other signs or symptoms of cardiac dysfunction. The incidence of definite ICI-myocarditis in our study is higher than previously reported, and if developed, it mostly presents within the first 5 administered doses.^21^, ^22^, ^23^ The median time to the occurrence of ICI-myocarditis after the first ICI dose was 40 days. Only 1 patient developed an ICI-myocarditis at a later instance, but this diagnosis could have been clinically masked by a prednisolone regimen given for concomitant ICI-hepatitis. Notably, higher hs-TnT levels were found in patients with definite ICI-myocarditis, compared to patients with only asymptomatic hs-TnT rise or high-probability of myocarditis (Table 1). All patients diagnosed with definite ICI-myocarditis had hs-TnT levels >160 ng/L at least once during ICI treatment, while the values of those with low and high ICI-myocarditis probability did not exceed 94 ng/L and 156 ng/L, respectively.

### Identifying patients at risk

Baseline characteristics of patients with and without elevated hs-TnT during our study follow-up are shown in Supplementary Table 1. Most patients who developed a definite ICI-myocarditis were male (75%) (Supplementary Table 2). Other patient demographics; age (63 ± 12 vs 69 ± 7 years), BMI (25 vs 25 kg/m^2^), systolic blood pressure (139 vs 122 mm Hg), estimated glomerular filtration rate (85 vs 87 ml/min/1.73 m^2^), and Hb (8.1 vs 8.0 mmol/L) did not significantly differ between the 2 groups. However, both baseline hs-TnT (15 ng/L; IQR: 12-39 ng/L vs 10 ng/L; IQR: 6-15 ng/L, *P* = 0.022) and N-terminal prohormone of B-type natriuretic peptide levels (1,639 ng/L; IQR: 450-12917 ng/L vs 138 ng/L; IQR: 52-336 ng/L, *P* = 0.019, respectively) were higher in patients that subsequently developed ICI-myocarditis.

To simultaneously evaluate the effect of several clinical factors on the development of ICI-myocarditis, we performed Cox-regression analyses. Variables used in the analysis were based on relationship significance illustrated in directed acyclic graph (Supplementary Figure 2). As shown in Table 4, hs-TnT doubling at the first visit demonstrated the most relevant association with ICI-myocarditis (HR 31.71 [95% CI: 5.91-170.23]).

**Table 4:** Cox-Regression Analysis: Unadjusted and Adjusted Risk Estimates for Doubling of hs-TnT Levels Between Baseline and Second Treatment Dose

	Model 1		Model 2	
	HR (95% CI)	*P* Value	HR (95% CI)	*P* Value
Doubling T2-BL	25.32 (5.62-114.15)	<0.001	31.71 (5.91-170.23)	<0.001
RCV T2-BL	12.68 (2.83-56.77)	0.001	10.43 (2.15-50.61)	0.004

Model 1: unadjusted.

Model 2: adjusted for diabetes and combination therapy (immune checkpoint inhibitor).

BL = baseline; hs-TnT = high-sensitive troponin T; RCV = reference change value; T2 = treatment dose 2.

### Immune-related adverse events (irAE) and patient survival

**Figure 1:**
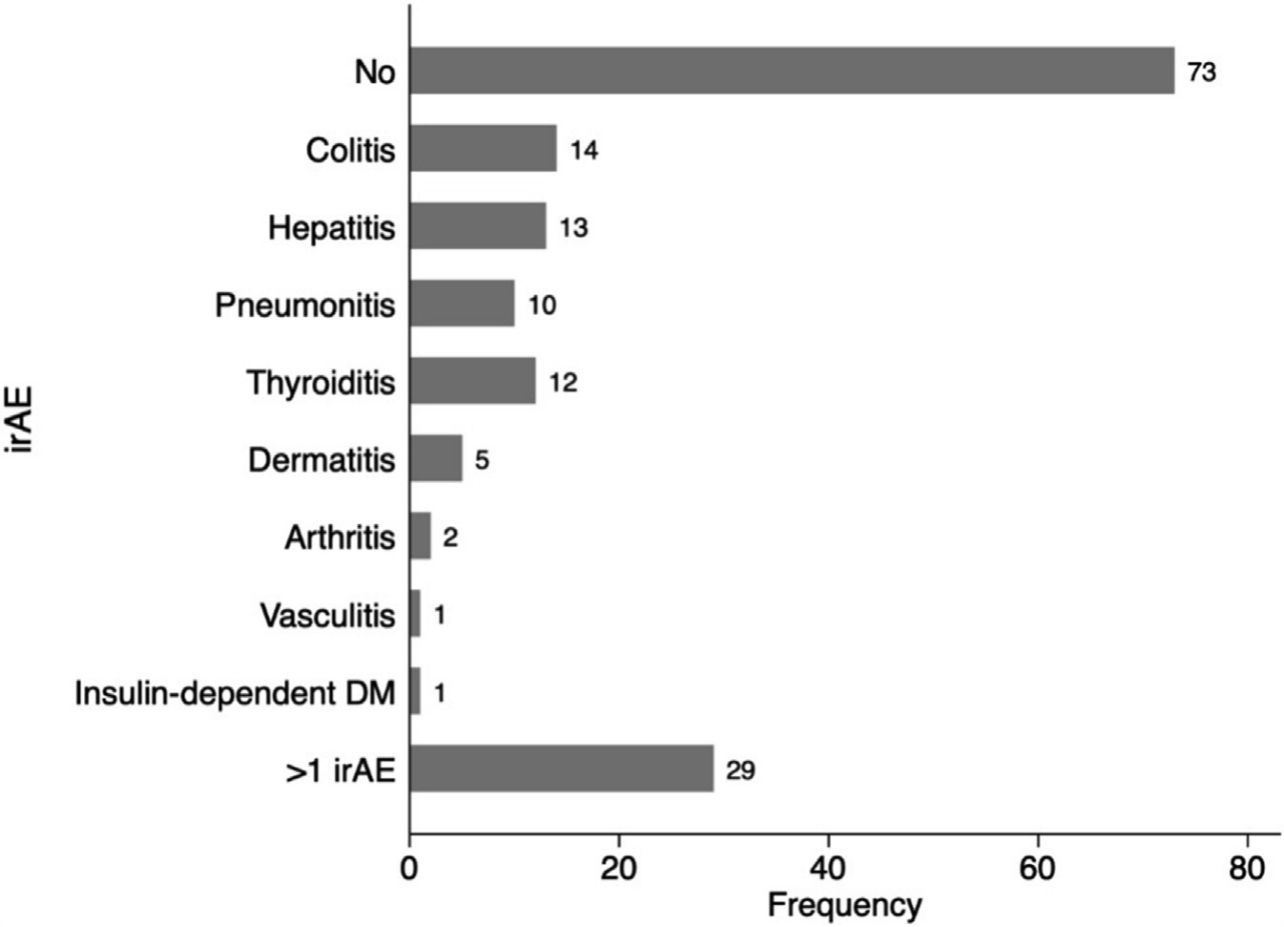
**Incidence of Other ICI-Related Adverse Effects During ICI Treatment** The figure presents an overview and the frequency of various ICI-related adverse effects throughout the course of ICI treatment. DM = diabetes mellitus; ICI = immune checkpoint inhibitor; iRAE = immune-related adverse event.

**Figure 2:**
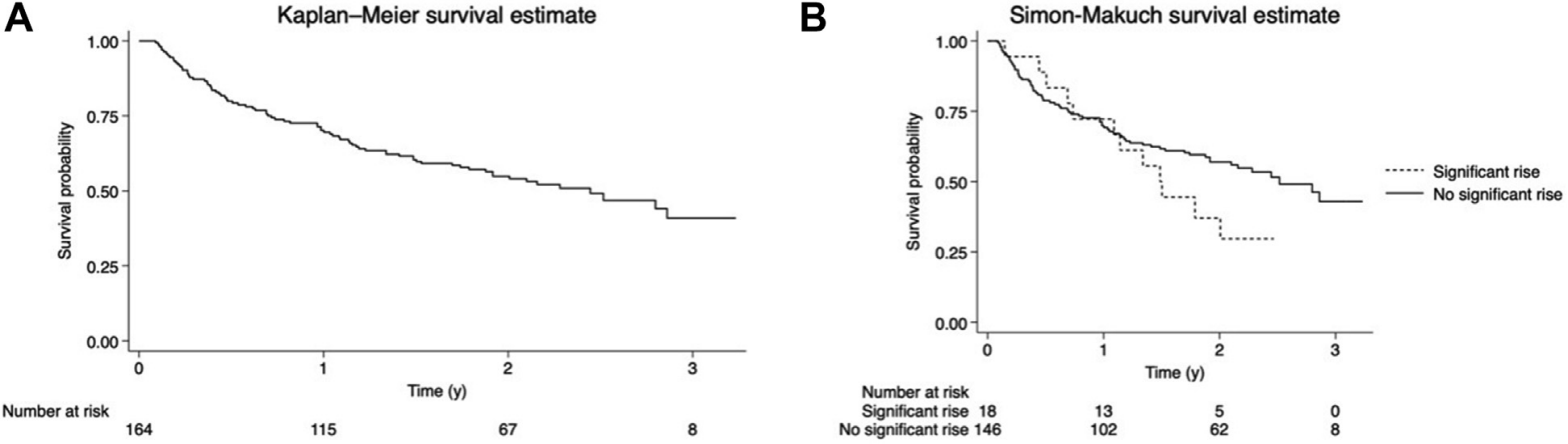
**Survival in Patient Population** (A) Overall survival (B) by occurrence of significant TnT rise. The figure depicts survival curves of patients undergoing immune checkpoint inhibitor (ICI) treatment, effectively illustrating the impact of significantly elevated hs-TnT on patient outcomes. hs-TnT = high-sensitivity troponin T.

In total, 91 patients (55%) developed irAE during ICI therapy, of whom the combination of 2 or more irAE was the most common (31%), followed by ICI-colitis (15%), ICI-hepatitis (14%), and ICI-thyroiditis (13%) (Figure 1). During our study follow-up with a mean duration of 1.60 (SD ± 0.91 years, 81 patients (49%) died (Table 5). Cancer progression was the most common cause of death (N = 73; 90%), while only 3 patients (4%) died due to a CV cause (Table 5). Only 1 of those patients was diagnosed with ICI-myocarditis, while the other 2 patients had low and high ICI-myocarditis probability. Patients with elevated hs-TnT demonstrated an overall worse survival regarding all-cause mortality (Figure 2).

**Table 5:** Mortality in Patient Population

No	83 (51)
Yes	81 (49)
CV	3 (4)
Cancer progression	73 (90)
Unknown	5 (6)

Values are n (%).

CV = cardiovascular.

## Discussion

This study represents one of the first and most comprehensive investigations into the serial hs-TnT monitoring during ICI treatment and its utility in identifying and diagnosing ICI-induced myocarditis (Central Illustration). Among patients receiving ICI treatment, a significant majority (58%) showed elevated hs-TnT levels (≥14 ng/L) at some point during their therapy, with 16% experiencing a doubling in hs-TnT levels (2 × ULN). Despite these elevations, most patients (70%) did not meet the criteria for a definitive diagnosis of ICI-induced myocarditis. Interestingly, a substantial number of patients with hs-TnT elevations were asymptomatic and showed no other signs of cardiac damage. Patients with a confirmed diagnosis of ICI-myocarditis exhibited significantly higher hs-TnT values than those with a lower probability of myocarditis. Despite a higher incidence of ICI-myocarditis observed in this study, overall cardiac mortality rate remained relatively low at 4%. This suggests that nonfulminant ICI-myocarditis is relatively common and that favorable outcomes may result from early detection and effective management strategies. It is important to acknowledge that the mortality rate associated with ICI-myocarditis can indeed vary significantly based on the severity of the condition. Fulminant myocarditis, characterized by a rapid and severe presentation of cardiac inflammation, is known to have a high mortality rate, up to 50%.^12^ However, the situation is more complex when we consider nonfulminant cases of ICI-myocarditis.^24^^,^^25^ One of the challenges in estimating the mortality rate for nonfulminant ICI-myocarditis is the high incidence of missed or underdiagnosed cases.

**Central Illustration:**
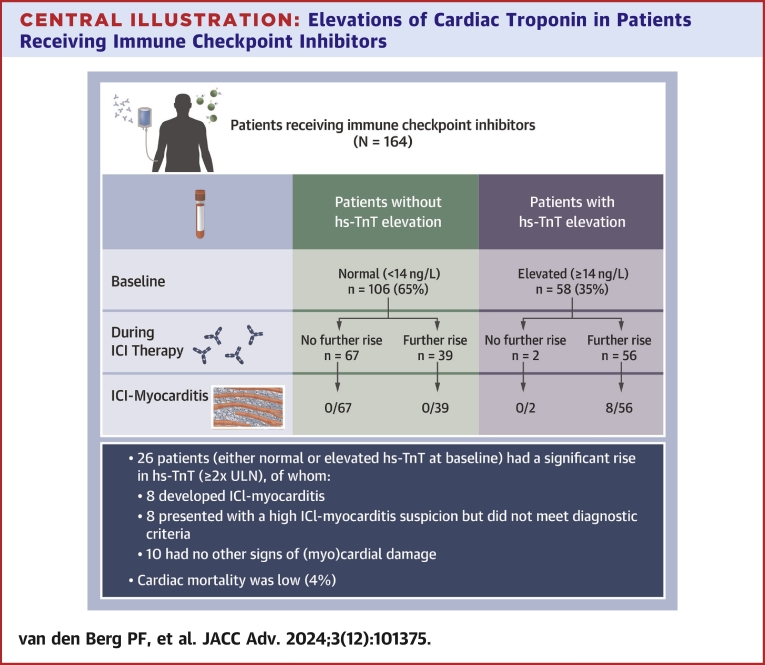
**Elevations of Cardiac Troponin in Patients Receiving Immune Checkpoint Inhibitors** Patients (N = 164) receiving immune checkpoint inhibitors (ICIs) were frequently monitored for their high-sensitivity troponin T (hs-TnT) values. The figure presents an overview of the number of patients (in %) who demonstrated elevated troponin values at baseline, during ICI therapy, and the number of patients diagnosed with ICI myocarditis.

The diagnostic role and increased incidence of elevated cardiac troponins in ICI-myocarditis has been well established in previous studies. Mahmood et al demonstrated that hs-TnT is one of the most sensitive biomarkers, showing hs-TnT elevations in 86% of patients that developed ICI-myocarditis. However, increased incidence of cardiac troponin elevations without definite ICI-myocarditis diagnosis has also been reported. Furukawa et al reported hs-TnI elevations in over 14% of patients, with only 4 cases necessitating immediate ICI discontinuation.^22^ Tamura et al^26^ further supported these findings, observing hs-TnI elevation in 18 patients, with only 6 of them being diagnosed with ICI-myocarditis. Furthermore, Waliany et al indicated that the positive associated value of hs-TnI was only 12.5% with a threshold of ≥55 ng/L, underscoring the ongoing clinical challenge in determining the specificity and sensitivity of cardiac biomarkers in diagnosing ICI-myocarditis.^27^ Our study provides further evidence that ICI-myocarditis is a rare complication, and most patients with elevated hs-TnT do not develop definite ICI-myocarditis. Nevertheless, ICI-myocarditis is still more prevalent (∼5%) than previously reported.

Despite the diagnostic complexity, our research reveals that serial hs-TnT measurements have important prognostic value. The European Society of Cardiology has recently published extensive guidelines regarding cardio-oncology, with hs-TnT playing a central role in the diagnosis and treatment of ICI-myocarditis.^20^ Unfortunately, these guidelines do not give clear recommendations on what change in hs-TnT should be considered significant and should trigger additional work-up. We have established that consistent low hs-TnT values (<100 ng/L) could effectively serve as a negative association marker, ruling out myocarditis in patients being treated with ICI. Conversely, in cases of definite myocarditis, hs-TnT levels consistently exceeded values of >160 ng/L. These findings highlight the potential of serial hs-TnT monitoring as a useful tool for assessing myocarditis risk and ruling out the condition in ICI-treated patients. Our results also indicate that the timing of the hs-TnT rise, with a doubling at the time of the second cycle being the best association, as well as the height of the hs-TnT levels (with values >100 ng/L being more associated) should be taken into account when making an ICI-myocarditis diagnosis.

One of the important observations in our study was that asymptomatic hs-TnT rises are very common in patients treated with ICI. *Hypertroponinemia*, as evidenced by only elevated hs-TnT levels, may represent one of distinct clinical entities that merits differentiation and distinct therapeutic approaches from low-grade and more aggressive forms of ICI-myocarditis. However, further research is needed to better understand the relevance and impact of these elevations in guiding clinical decisions during ICI treatment.

Finally, despite the notable occurrence of presumed cardiac injury, as evidenced by elevated hs-TnT levels and a higher frequency of ICI-myocarditis in our study compared to prior reports, the overall cardiac mortality remained relatively low. Nevertheless, it is imperative to highlight those individuals exhibiting significantly elevated hs-TnT values who demonstrated poorer clinical outcomes in our investigation regarding all-cause mortality. While we may argue that timely withdrawal of ICI treatment in patients with hs-TnT elevations, with or without the start of immunosuppressive therapy could reduce the incidence of severe cardiac complications, this should always be weighed against the most optimal cancer treatment. Given the long half-life of immunotherapy agents, temporarily delaying immunotherapy to serially evaluate the cause and course of hs-TnT elevations, especially in older patients with high CV morbidity, is likely a safe approach. In a retrospective study of 35 patients who developed ICI-myocarditis, a longer time interval to starting steroids was correlated with a higher incidence of major adverse cardiac events.^22^ Therefore, a prompt start of immunosuppressive therapy next to the ICI withdrawal is warranted in patients with a high likelihood of ICI-myocarditis and should be started without delay in patients with definite ICI-myocarditis. In contrast, there is likely no role for immunosuppressive treatment in patients with hypertroponinemia only without presence of other minor or major criteria. For these patients, close surveillance with/without interrupting ICI therapy and close monitoring of hs-TnT to determine its course together with assessment of new symptoms and ECG changes is likely a safe approach, especially when hs-TnT elevations develop at later cycles of ICI therapy and absolute hs-TnT levels remain <100 ng/L. Finally, we strongly urge clinicians to make multidisciplinary team decisions where the risk and benefits of ICI treatment and treatment discontinuation are weighted for each patient individually.

The findings from this study may contribute to the development of evidence-based guidelines and risk stratification strategies for clinicians in the cardio-oncology field, enabling them to identify patients who would benefit from closer cardiac monitoring, prompt intervention, and tailored cardio-protective strategies during ICI treatment.

### Study limitations

Some limitations of this study merit mentioning. The nature of this observational prospective study does not allow to prove causation but rather demonstrates associations. The small sample size warrants caution when interpreting the presented findings, as underpowering may have influenced the outcomes. hs-TnT levels differ between male and female patients. In this relatively small study, we lacked the power to perform robust sex-specific subanalyses, which we acknowledge is a limitation. Due to different treatment protocol and therefore lack of baseline hs-TnT measurements, only 2 patients with lung cancer were included in our study. Furthermore, while we excluded all patients that tested positive for COVID, influence of subclinical and undiagnosed COVID infection on hs-TnT elevations in our study population could not be excluded. Given the small number of outcomes in this study and the limited number of covariates in our model, we emphasize that the conclusions in this study warrant careful interpretation.

## Conclusions

The findings of our study provide evidence for a higher incidence of ICI-myocarditis and a lower rate of cardiac mortality than previously reported, thus highlighting the importance of proactive measurements of hs-TnT in patients undergoing ICI treatment. Additionally, we demonstrate that a majority of patients who undergo ICI treatment exhibit elevated hs-TnT levels and frequently experience no other signs or symptoms of cardiac injury. *Hypertroponinemia* may be a novel and separate clinical phenomenon that requires a more conservative diagnostic work-up and treatment approach than ICI-myocarditis. Although the specific pathophysiological mechanisms remain unclear, our findings indicate that invasive clinical testing and proactive anti-inflammatory treatment may not be required in patients with hypertroponinemia.

PERSPECTIVES **COMPETENCY IN PATIENT CARE AND PROCEDURAL SKILLS:** This study highlights the importance of hs-TnT in managing ICI-myocarditis. Regular hs-TnT monitoring allows for early detection and risk stratification, facilitating timely intervention to prevent severe outcomes. For patients with minor, asymptomatic hs-TnT increases, a conservative approach is suggested, avoiding invasive diagnostics. After 6 to 8 ICI cycles, serial hs-TnT measurements can be discontinued due to reduced myocarditis risk. In cases of symptomatic significant hs-TnT rises (>100 ng/L), immediate diagnostics, ICI-therapy discontinuation, and steroid treatment are warranted.

**TRANSLATIONAL OUTLOOK:** Future research should refine hs-TnT change criteria for better diagnostic specificity and explore hypertroponinemias’ mechanisms and implications. The role of managing elevated hs-TnT levels is crucial in developing and adjusting current guidelines for cardiac monitoring during ICI therapy.

## Funding support and author disclosures

This work was supported by a grant from the 10.13039/501100000781European Research Council (ERC CoG 818715, SECRETE-HF). Support was received from grants from the Netherlands Heart Foundation (CVON SHE-PREDICTS-HF, grant2017-21; CVON RED-CVD, grant 2017-11; CVON PREDICT2, grant 2018-30; and CVON DOUBLE DOSE, grant 2020B005) and by a grant from the 10.13039/501100001674LeDucq Foundation (Cure PhosphoLambaN induced Cardiomyopathy [Cure-PLaN]). Dr Meijers is supported by the Mandema-Stipendium of the Junior Scientific Masterclass 2020-10 of the 10.13039/501100005075University Medical Center Groningen and by the Dutch Heart Foundation (Dekker grant 03-005-2021-T005). The UMCG, which employs/employed several of the authors, has received research grants and/or fees from AstraZeneca, Abbott, Boehringer Ingelheim, Cardior Pharmaceuticals GmbH, Novo Nordisk, and Roche; Dr de Boer has had speaker engagements with and/or received fees from and/or served on an advisory board for Abbott, AstraZeneca, Bristol Myers Squibb, Cardior Pharmaceuticals GmbH, NovoNordisk, and Roche; and received travel support from Abbott, Cardior Cardior Pharmaceuticals GmbH, and NovoNordisk. Dr van der Meer has received grant support or consultancy fees from Novartis, Pharma Nord, Pfizer, Ionis, Astra Zeneca, Vifor Pharma, Pharmacosmos, BridgeBio, and NovoNordisk. All other authors have reported that they have no relationships relevant to the contents of this paper to disclose.
