# Supplementary material for: Elevations of Cardiac Troponin in Patients Receiving Immune Checkpoint Inhibitors: Data From a Prospective Study
Source: JACC Adv. 2024 Nov 7;3(12):101375. doi: 10.1016/j.jacadv.2024.101375 (PMC11584941; doi:10.1016/j.jacadv.2024.101375)
Supplement: Supplementary Material [file mmc1.pdf]

Supplementary Figure 1. Flow-diagram of study population.

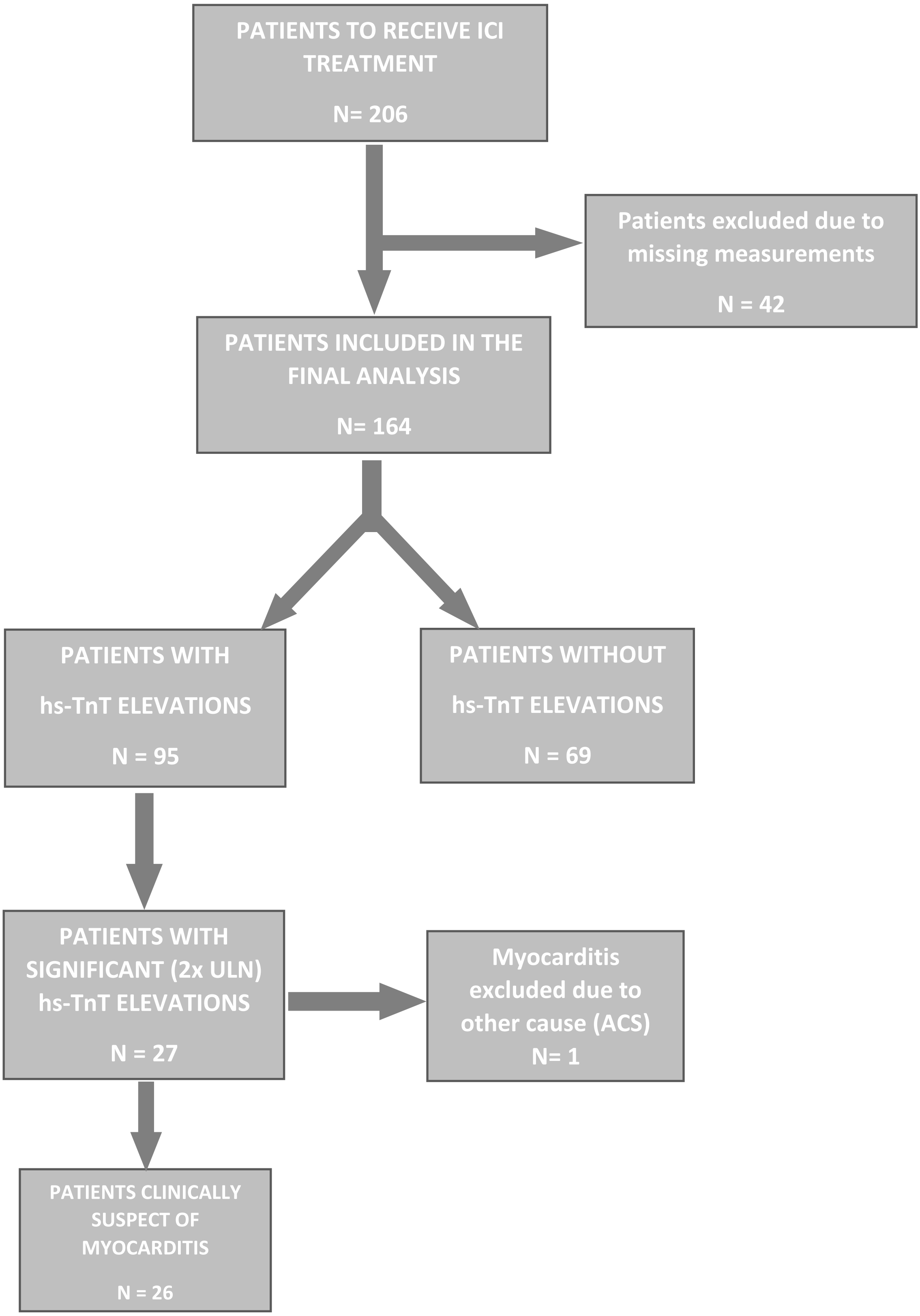

Supplementary Figure 2. Directed acyclic graph (DAG) to determine multivariable regression.

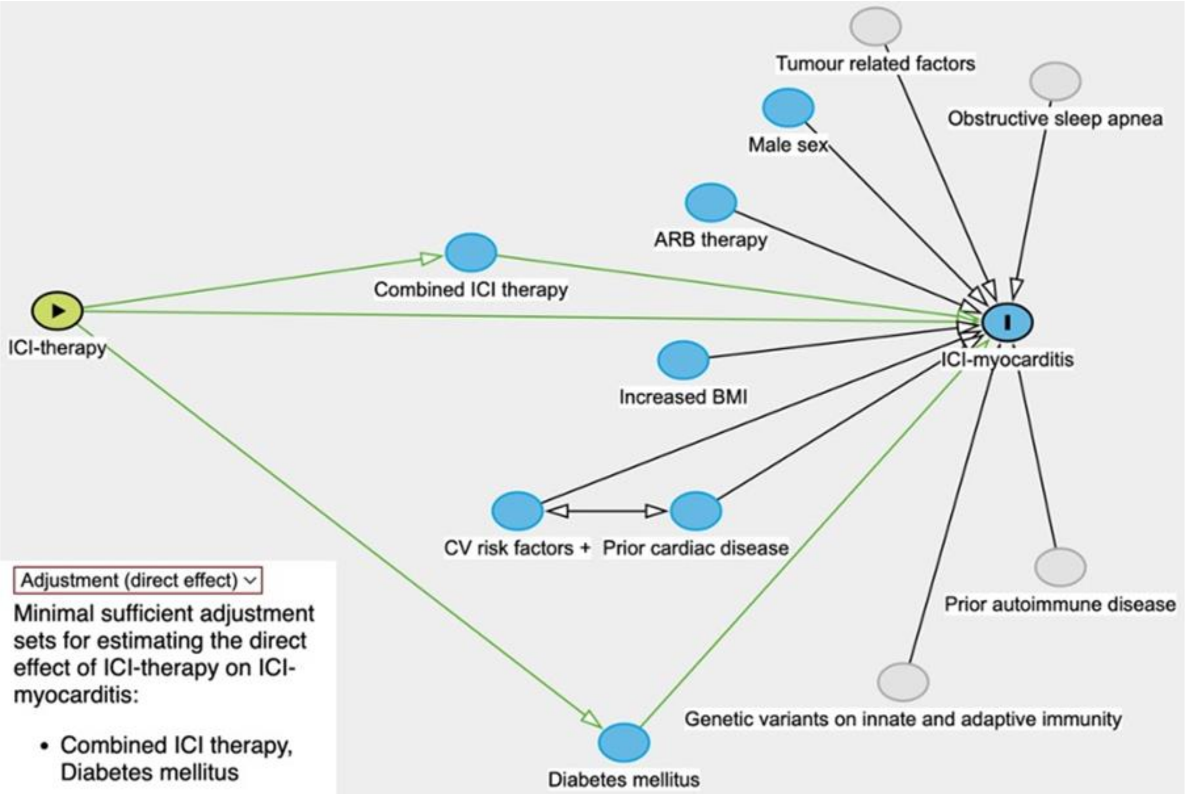

Supplementary Figure 3. High- sensitive troponin T (Hs-TnT) trajectories during ICI-therapy.

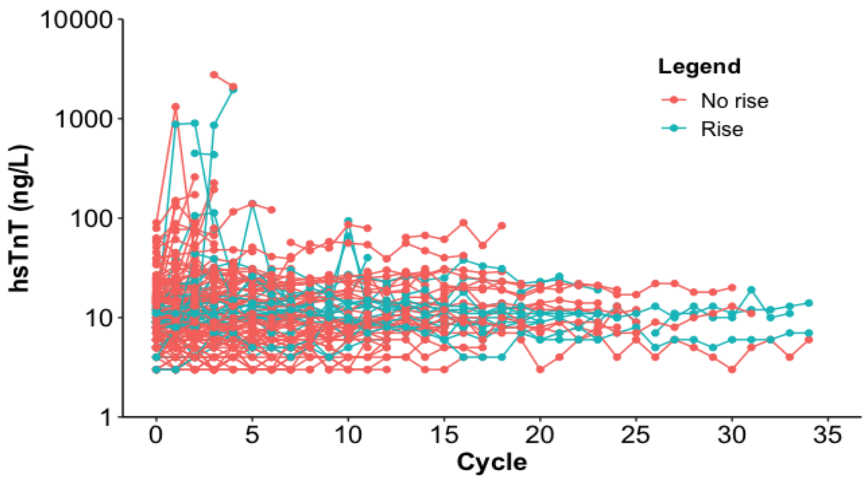

Supplementary Table 1. Baseline characteristics of patients with and without elevated hs-TnT levels (*i.e.* ≥14 ng\L) during ICI treatment.

Factor	Hs-TnT <14 ng/L N = 69	Hs-TnT ≥14 ng/L N = 95	p-value
Patient demographics			
Gender			<0.001
Male (%)	34 (49)	70 (74)	
Female (%)	35 (51)	25 (26)	
Age, (SD), years	57 ± 13	67 ± 8	<0.001
BMI (IQR), kg/m^2	24.1 (21.8-27.0)	26.0 (23.3-28.5)	0.090
SBP (IQR), mmHg	135 (125, 140)	140 (130, 151)	0.35
DBP (IQR), mmHg	80 (74, 90)	80 (70, 88)	0.40
Laboratory parameters			
hsTnT (IQR), ng/L	6 (5, 9)	14.5 (11, 20)	<0.001
NT-proBNP (IQR), ng/L	75 (44, 161)	297 (62, 784)	0.054
eGFR (IQR), ml/min/1,73 m2	92 (84, 101)	87 (69-93.5)	<0.001
Oncological demographics			
Type of cancer (%)			0.031
Renal	6 (9)	24 (26)	
Melanoma	52 (75)	45 (47)	
Lung	1 (1)	1 (1)	
Head and neck	2 (2)	6 (6)	
Bladder	3 (4)	7 (7)	
Merkel cell carcinoma	0 (0)	2 (2)	
Squamous cell skin	2 (2)	5 (5)	
GI	3 (4)	5 (5)	
Active cancer treatment (%)			0.20
PD1	34 (49)	37 (38)	
PDL1	7 (10)	20 (21)	
CTLA4	1 (1)	0 (0)	
PD/PDL1 + CTLA4	24 (35)	33 (35)	
ICI + TKI	2 (3)	5 (5)	
ICI + other	1 (1)	0 (0)	
Cardiovascular history			
CV history (%)			<0.001
No	64 (93)	51 (55)	
Yes	5 (7)	44 (45)	
Risk factors (%)			0.002
No	20 (29)	11 (12)	
Yes	43 (62)	78 (82)	
Unknown	6 (9)	6 (6)	

Abbreviations: BMI, body mass index; SBP, systolic blood pressure; DBP, diastolic blood pressure; HR, heart rate; hsTnT, high-sensitivity troponin T; NT-proBNP, N-terminal prohormone of B-type natriuretic peptide; eGFR, estimated glomerular filtration rate; Hb,

hemoglobin; TKI, tyrosine kinase inhibitor; PD1, programmed cell death protein 1; PDL1, programmed death-ligand 1; CTLA4, cytotoxic T-lymphocyte associated protein 4; ICI, immune checkpoint inhibitor; CV, cardiovascular; ACS, acute coronary syndrome; CAD, coronary artery disease; DVT, deep venous thromboembolism; PE, pulmonary embolism; TIA transient ischemic attack; CVA, cerebrovascular accident; ACEi, angiotensin converting enzyme inhibitor; ARB angiotensin receptor blocker; VKA, vitamin K antagonist; NOAC, novel oral anticoagulant; LMWH, low molecular weight heparin.

Supplementary Table 2. Baseline characteristics of patient population by myocarditis status.

Factor	No myocarditis (N=156)	Myocarditis (N=8)	p-value
Patient demographics			
Gender (%)			0.49
Male	98 (63)	6 (75)	
Female	58 (37)	2 (25)	
Age, (SD), years	63±12	69±7	0.15
BMI (IQR), kg/m^2	25.1 (22.6-28.0)	25.1 (22.1-28.2)	0.94
SBP (IQR), mmHg	139 (130, 147)	122 (100, 144)	0.50
DBP (IQR), mmHg	80 (70, 90)	71.5 (60, 83)	0.47
HR (IQR), bpm	83 (67, 94)	91 (66, 137)	0.39
Laboratory parameters			
Hs-TnT (IQR), mmol/L	10 (6, 15)	15 (12, 39)	0.022
NT-proBNP (IQR), mmol/L	138 (52, 336)	1639 (450, 12917)	0.019
eGFR (IQR), ml/min/1,73 m^2	85 (66, 94)	87 (69, 93.5)	0.80
Hb (IQR), mmol/L	8.1 (6.9, 8.9)	8.0 (7, 9.6)	0.52
Leucocytes (IQR), mmol/L	7.6 (6.1, 9.8)	7.7 (4.5, 10.6)	0.60
Oncological demographics			
Cancer type (%)			0.15
Renal	28 (18)	2 (25)	
Melanoma	92 (59)	5 (62)	
Lung	1 (0.6)	1 (12)	
Head and neck	8 (5)	0 (0)	
Bladder	10 (6)	0 (0)	
Merkel cell carcinoma	2 (1)	0 (0)	
Squamous cell skin	7 (4)	0 (0)	
GI	8 (5)	0 (0)	
Active cancer treatment (%)			0.97
PD1	67 (43)	3 (37)	
PDL1	25 (16.	2 (25)	
CTLA4	1 (0.5)	0 (0)	
PD/PDL1 + CTLA4	55 (35)	3 (37)	
ICI + TKI	7 (4)	0 (0)	
ICI + other	1 (0.5)	0 (0)	
Cardiovascular history			
CV history (%)			0.45
No	112 (72)	4 (50)	
Yes	44 (28)	4 (50)	
Risk factors (%)			0.066
No	30 (19)	2 (25)	
Yes	114 (74)	6 (75)	
Unknown	12 (8)	0 (0)	

Abbreviations: BMI, body mass index; SBP, systolic blood pressure; DBP, diastolic blood pressure; HR, heart rate; hsTnT, high-sensitivity troponin T; NT-proBNP, N-terminal

prohormone of B-type natriuretic peptide; eGFR, estimated glomerular filtration rate; Hb, hemoglobin; TKI, tyrosine kinase inhibitor; PD1, programmed cell death protein 1; PDL1, programmed death-ligand 1; CTLA4, cytotoxic T-lymphocyte associated protein 4; ICI, immune checkpoint inhibitor; CV, cardiovascular; ACS, acute coronary syndrome; CAD, coronary artery disease; DVT, deep venous thromboembolism; PE, pulmonary embolism; TIA transient ischemic attack; CVA, cerebrovascular accident; ACEi, angiotensin converting enzyme inhibitor; ARB angiotensin receptor blocker; VKA, vitamin K antagonist; NOAC, novel oral anticoagulant; LMWH, low molecular weight heparin.

Supplementary Table 3. Clinical characteristics of patients at the time of definite ICI myocarditis diagnose.

N	Hs-TnT max (ng/L)	CK max (U/L)	eGFR (ml/min *1.73m^2)	NTproBNP max (ng/L)	LVEF during treatment (%)	cMRI myocardial oedema (T2)	cMRI nonischaemic myocardial injury (LGE/ ECV/ T1)
1	1382	39	47	19266	27	-	+
2	146	31	51	27165	30	-	+
3	882	512	95	51	63	-	+
4	134	104	84	1268	>60	+	+
5	210	-	94	182	>60	-	+
6	199	-	90	359	>60	+	+
7	3395	1787	29	9738	>60	-	+
8	249	46	92	256	>60	-	+

Hs-TnT rise is a difference between highest hs-TnT value and baseline value.

Patient 7 died of sudden cardiac death due to ICI- myocarditis.

Abbreviations: LVEF, left ventricular ejection fraction; hs-TnT, high sensitive troponin T; CK, creatin kinase; eGFR, estimated glomerular filtration rate; NT-proBNP, N-terminal prohormone of B-type natriuretic peptide; cMRI, cardiac magnetic resonance image; LGE, late gadolinium enhancement.
